# Predicting Finite-Bias Tunneling Current Properties from Zero-Bias Features: The Frontier Orbital Bias Dependence at an Exemplar Case of DNA Nucleotides in a Nanogap

**DOI:** 10.3390/nano11113021

**Published:** 2021-11-10

**Authors:** Ivana Djurišić, Vladimir P. Jovanović, Miloš S. Dražić, Aleksandar Ž. Tomović, Radomir Zikic

**Affiliations:** Institute for Multidisciplinary Research, University of Belgrade, Kneza Višeslava 1, 11030 Belgrade, Serbia; ivana.djurisic@imsi.bg.ac.rs (I.D.); vladimir.jovanovic@imsi.rs (V.P.J.); milos.drazic@imsi.bg.ac.rs (M.S.D.); aleksandar.tomovic@imsi.bg.ac.rs (A.Ž.T.)

**Keywords:** electronic transport, single-molecule, DNA and protein sequencing, molecular level pinning, electrostatic potential, DFT+NEGF

## Abstract

The electrical current properties of single-molecule sensing devices based on electronic (tunneling) transport strongly depend on molecule frontier orbital energy, spatial distribution, and position with respect to the electrodes. Here, we present an analysis of the bias dependence of molecule frontier orbital properties at an exemplar case of DNA nucleotides in the gap between H-terminated (3, 3) carbon nanotube (CNT) electrodes and its relation to transversal current rectification. The electronic transport properties of this simple single-molecule device, whose characteristic is the absence of covalent bonding between electrodes and a molecule between them, were obtained using density functional theory and non-equilibrium Green’s functions. As in our previous studies, we could observe two distinct bias dependences of frontier orbital energies: the so-called strong and the weak pinning regimes. We established a procedure, from zero-bias and empty-gap characteristics, to estimate finite-bias electronic tunneling transport properties, i.e., whether the molecular junction would operate in the weak or strong pinning regime. We also discuss the use of the zero-bias approximation to calculate electric current properties at finite bias. The results from this work could have an impact on the design of new single-molecule applications that use tunneling current or rectification applicable in high-sensitivity sensors, protein, or DNA sequencing.

## 1. Introduction

In the last-generation protein and DNA sequencing devices, the chain-like molecule, driven by an electrophoretic field, translocates through a nanopore [1,2,3,4]. In a subclass of these devices, side-embedded transversal electrodes are placed around the nanopore, and the tunneling current through a single chain monomer is measured for sequence readout [5,6,7,8,9,10]. Experimental realizations of such setups were performed using solid-state nanopores [6,7,11]. Nanopores could be realized in various membrane materials, such as silicon oxide, silicon nitride, graphene, and other 2D materials [2,3,6,7,11]. Proposals for transversal electrode materials range from gold [12,13], graphene [3,9,14,15,16], or carbon nanotubes [8,17,18] to complex, which include heterostructures [19] or molecular functionalization [20,21]. The functionalization of electrodes could be beneficial for transversal electronic transport, as it may increase tunneling currents and lower the operating bias of devices [14,17,18,21].

In a configuration where side-embedded transversal electrodes are placed around a nanopore, the probability of finding covalent bonding and, therefore, the strong coupling between the molecule and the nano-sized electrodes is almost negligible. As the tunneling current depends primarily on the strength of coupling between the molecule and the electrodes [22], in such molecular junction devices, the electric current is generally low compared to single-molecule rectifiers (molecule usually covalently bound to electrodes) [23,24,25,26,27,28]. The exception may occur when molecular levels energy enter “the bias window” (the energy range between electrochemical potentials of electrodes) and begin contributing to the electronic transport and increasing the current. Commonly, only a few levels around Fermi energy, whose wave function is mainly concentrated on the molecule, may participate in the transport and, in most cases, include frontier orbitals HOMO and LUMO (highest occupied and lowest unoccupied molecular orbitals).

We have previously shown, using density functional theory (DFT) and non-equilibrium Green’s functions (NEGF), the existence of two different regimes of bias dependence of the frontier orbitals in molecular junctions: the strong and the weak pinning [18,29]. The former refers to the classical meaning of pinning: bias variations of the molecular orbital energy and the electrochemical potential of anelectrode are the same. In the latter regime, these variations are not the same. The weak to the strong pinning regime transition will occur when the molecular frontier orbital hybridizes and overlaps in energy and real space, with Bloch states from only one electrode. At that bias value, the accumulation of charge at the molecule appears, shifting the orbital energy and pushing it away from the bias window, thus lowering the tunneling current and raising its rectification.

In this work, we study the bias dependence of the frontier orbitals of DNA nucleotides in the gap between two H-terminated (3, 3) carbon nanotubes (CNTs), employing DFT coupled with NEGF. We found the existence of both the strong and the weak regimes and investigate how the empty-gap and zero-bias properties could be utilized to predict finite-bias current and its rectification. We shall also discuss the applicability of the zero-bias approximation for the electronic transport properties estimation and compliance with a set of rules for building molecular rectifiers [30,31].

We note here that the frontier orbitals are not addressed as orbitals of isolated molecules or the HOMO and LUMO of the molecular junction as a whole. In fact, they aremolecular projected self-consistent Hamiltonian (MPSH) HOMO and LUMO. That means that the atomic orbitals that create the isolated molecule HOMO/LUMO have the highest contribution to molecular junction MPSH HOMO/LUMO. In addition, we are aware that the results obtained for high bias values may not have practical application. However, we still presented those results for the sake of clarity of reasoning. From the experimental standpoint, the system consisting of DNA nucleotide between two H-terminated (3, 3) CNTs is feasible, as already extensively discussed in our previous work [18].

## 2. Computational Details

**Figure 1:**
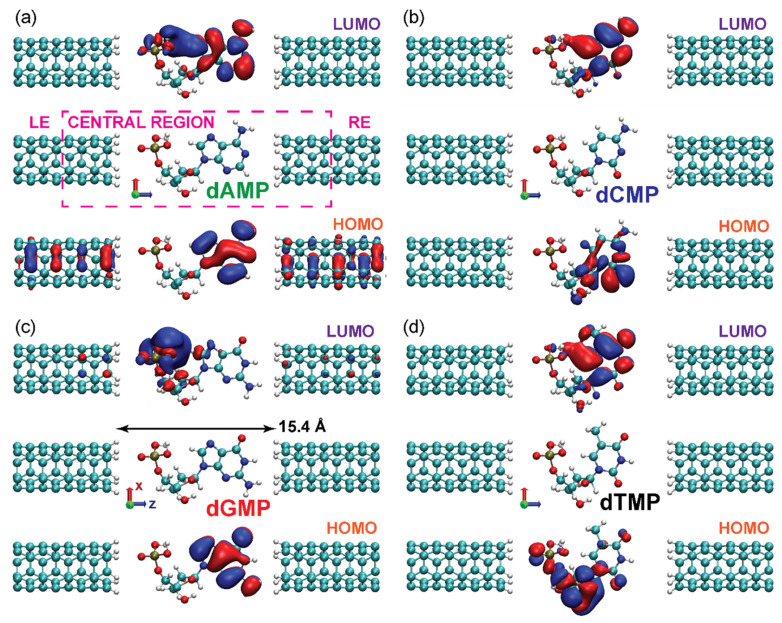
Deoxyribonucleotides (**a**) dAMP, (**b**) dCMP, (**c**) dGMP, and (**d**) dTMP between two H-terminated (3, 3) CNTs with 15.4 Å gap, i.e., the distance between H atoms of the left and the right tube, indicated by the black arrow in (**c**). The isosurfaces of the MPSH frontier orbital wave functions, HOMO and LUMO, calculated at zero bias, with the isovalues *ψ*_HOMO/LUMO_ = –0.05 (blue) and *ψ*_HOMO/LUMO_ = 0.05 (red). The central region, the left, and the right bulk electrode for the finite-bias calculations are marked with the dashed magenta rectangle, LE, and RE in (**a**). Red, green, and blue arrows indicate *x*, *y*, and *z* axes, respectively.

The relaxed geometries of four DNA nucleotides (deoxyadenosine monophosphate (dAMP), deoxycytidine monophosphate (dCMP), deoxyguanosine monophosphate (dGMP), and deoxythymidine monophosphate (dTMP)) and the hydrogen termination of CNTs were obtained from the SIESTA package (Figure 1) [32]. The tunneling transport simulations at finite bias were performed in TranSIESTA [33]. Two CNT unit cells, with the lattice constant of 2.468 Å, and a radius of 2.041 Å, were taken for the bulk electrode calculations [34]. In Figure 1, LE and RE denote the left and the right bulk electrode, respectively; the central region is found within the dashed orange rectangle, while the black arrow marks the width of the gap between CNTs. Other relevant parameters for the computation were 1 × 1 × 64 k-points for the electrode and 1 × 1 × 1 k-points (Γ-point) for the scattering region, a double zeta polarized basis set for all atoms, the real-space grid mesh cutoff of 200 Ry, and the Perdew–Burke–Ernzerhof (PBE) exchange-correlation functional [35]. Core electrons were described with Troullier–Martins norm-conserving pseudopotentials [36], and the atomic charge excess was obtained from the Hirshfeld population analysis. For the orbital visualization, VMD was used [37], while the post-processing tool for the extraction of the data and its analysis was SISL [38].

Since our TranSIESTA calculations are limited to the Γ-point, the *i*th MPSH orbital wave function *ψ_i_* has a simplified form:

(1) $$\psi_{i}(r)=\sum_{\mu }c_{\mu i}\phi_{\mu }(r),$$

where **r** is a vector of a point in real space, and *c_μi_* are the coefficients of a linear combination of atomic orbitals *φ_μ_*. In Figure 1, we plotted for all nucleotides the real-space isosurfaces of MPSH HOMO and LUMO wave functions, whose value is *ψ*_HOMO/LUMO_ = –0.05 (blue) and *ψ*_HOMO/LUMO_ = 0.05 (red). Such high isovalues are chosen to emphasize the atoms with the main contribution to the wave functions.

In TranSIESTA, the tunneling current *I* at finite bias *V* is obtained from the following equation:

(2) $$I(V)=\frac{2e}{h}\int_{-\infty }^{+\infty }dE\left[f(E-\mu_{L})-f(E-\mu_{R})\right]T(E,V),$$

where *f*(*E* − *μ_L/R_*) is the Fermi–Dirac function of the left/right electrode, *μ_L/R_* = *E*_F_ ± *eV*/2 is their electrochemical potentials, *E*_F_ is the Fermi energy, and *T*(*E*,*V*) is the transmission spectrum. Zero-bias approximation assumes that at low bias, the transmission spectrum is the same as at zero bias, i.e., *T*(*E*,*V*) = *T*(*E*,0). Therefore, to obtain finite-bias tunneling current in this approximation, only transmission spectrum *T*(*E*,0) calculation at zero bias is needed.

## 3. Results

### 3.1. Empty Gap Properties and the Weak Pinning

**Figure 2:**
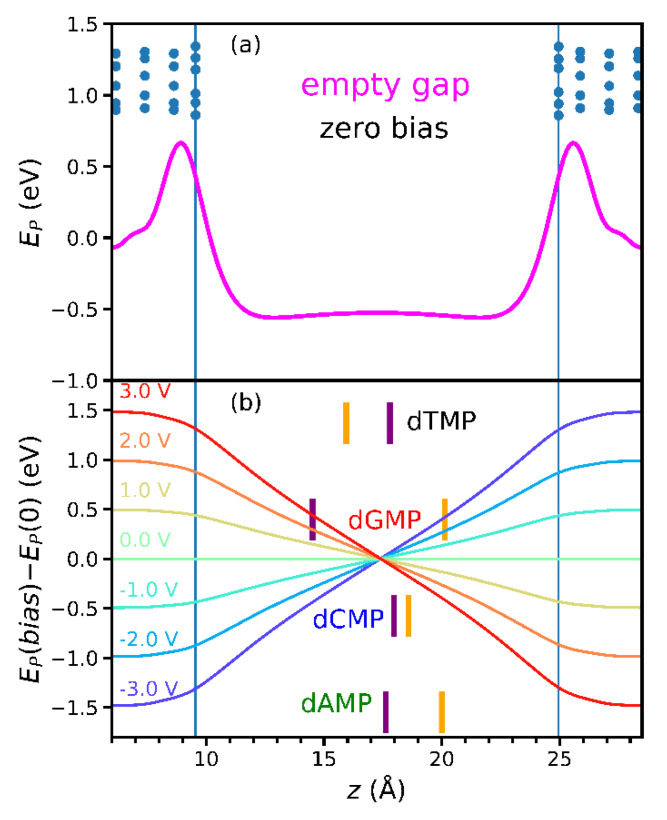
(**a**) The zero-bias electrostatic potential energy *E_P_* of an empty gap along the midline in the *z*-direction. Dots indicate atoms of CNTs, while vertical blue lines denote the gap edges. (**b**) The electrostatic potential energy *E_P_*(*bias*) − *E_P_*(0) of an empty gap along the midline in the *z*-direction for several bias values is indicated on the left. Vertical orange and purple lines are the average *z* coordinates of atoms having the most significant contributions to the nucleotide frontier orbitals HOMO and LUMO.

In the weak pinning (WP) regime, the bias dependence of (frontier) orbital energies is driven mainly by the *empty* gap electrostatic potential energy *E_P_* and their spatial positions in the gap [29]. Figure 1 presents the spatial distribution of frontier orbitals of four DNA nucleotides. The HOMO orbital of both dAMP and dGMP occupies the nucleobase part of the molecule (Figure 1a,c). Similarly, for both dCMP and dTMP, the LUMO orbital primarily occupies the nucleobase part (Figure 1b,d). At zerobias, the *E_P_* along the midline in the *z*-direction in an empty gap between H-terminated CNTs is given in Figure 2a, whose shape (magenta line) originates from the dipoles at CNT terminations [39,40]. Figure 2b shows the empty-gap electrostatic potential energy *E_P_*(*bias*) − *E_P_*(0) along the same line at different bias values and average *z* coordinates of HOMO and LUMO (<*z*_HOMO_> and <*z*_LUMO_>, orange and purple vertical lines, respectively, and also Figure 1) of nucleotides. Expectedly, the <*z*_HOMO_> values for dAMP and dGMP are close, owing to similar distribution of the HOMO orbitals (Figure 1a,c and Figure 2b). The same is true in the case of <*z*_LUMO_> for dCMP and dTMP (Figure 1b,d and Figure 2b). The LUMO orbital of dAMP spreads across the whole molecule, and, as a result, its <*z*_LUMO_> value is close to that of dCMP and dTMP (Figure 1a and Figure 2b). Thus, the orbital energy should vary linearly with bias, and this variation should be the largest for orbitals localized farthest away from the gap center in the WP regime. The energy of orbitals located right (left) to the gap center will decrease (increase) with the bias rise. According to Figure 2b, the LUMO energy of all nucleotides should be nearly independent of bias, except for dGMP, for which it should increase at a rate of 0.15 eV/V. The HOMO energy should decrease for all studied molecules but dTMP, for which the rise is expected in the WP regime.

### 3.2. Finite-Bias Properties of the Nucleotide Molecular Junction

**Figure 3:**
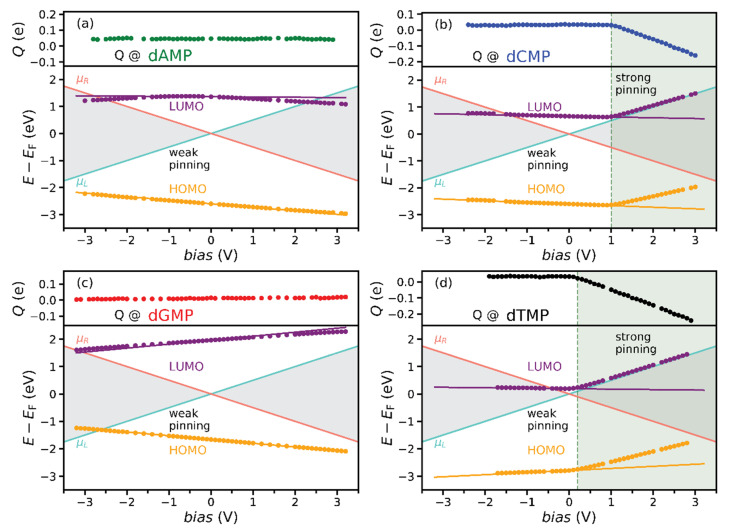
Pinning regimes diagram showing the bias dependence MPHS HOMO (orange dots) and LUMO (purple dots) energies with respect to Fermi energy *E*_F_ of (**a**) dAMP, (**b**) dCMP, (**c**) dGMP, and (**d**) dTMP and Hirshfeld charge excess *Q* (black dots) accumulated on the molecules (in the upper part). Solid orange and purple lines are the weak-pinning HOMO and LUMO energy estimate *E*^WP^_HOMO/LUMO_(*bias*) = *E_P_*(*bias*, <*z*_HOMO/LUMO_>) − *E_P_*(0, <*z*_HOMO/LUMO_>) + *E*_HOMO/LUMO_(0) − *E*_F_ from the empty gap (Figure 2b). The green-shaded region marks the strong pinning bias domain, which starts at a bias value indicated with the green dotted line. The electrochemical potentials *μ_L_* and *μ_R_* of the left and the right electrode are given as light blue and red lines, and the bias window between them is the gray-shaded area.

Next, we plot in Figure 3 the evolution with the applied voltage of MPSH HOMO (orange dots) and LUMO (purple dots) energies *E*_HOMO/LUMO_(*bias*) − *E*_F_ with respect to the Fermi energy *E*_F_ of nucleotides between CNTs, as obtained from finite-bias TranSIESTA calculations. Frontier orbital energies are in the WP regime for most of the studied voltages. As anticipated, they rather well follow the linear weak-pinning frontier orbital energy estimate

*E*^WP^_HOMO/LUMO_(*bias*) *= E_P_*(*bias,* <*z*_HOMO/LUMO_>) − *E_P_*(0, <*z*_HOMO/LUMO_>) + *E*_HOMO/LUMO_(0) − *E*_F_, (3)

(orange and purple lines referring to HOMO and LUMO in Figure 3) from the empty gap in Figure 2b. The “empty-gap estimate” includes the averaging of only the *z* coordinates of atoms for which the atomic orbital coefficients *c*, from Equation (1), surpass some value (0.1 in this case). Therefore, the slight deviations that appear, as for dAMPLUMO, may be attributed to those simplifications. Some nucleotides, such as dAMP and dGMP, do not show signs of the strong pinning (SP), while the other molecules, dTMP and dCMP, enter the SP regime at positive bias values (Figure 3). At those voltages, 0.2 V for dTMP and 1 V for dCMP, both HOMO and LUMO energies of both nucleotides deviate from the WP lines. Simultaneously, the charge *Q* (Figure 3), obtained from the Hirshfeld population analysis, begins to accumulate on the molecule, causing the strong pinning of the orbitals to the electrochemical potential *μ_L_* of the left electrode (light blue line in Figure 3) [39,40].

### 3.3. Estimation of FiniteBias from the Empty Gap and Zero-Bias Properties

How to estimate, from zero-bias properties, whether the pinning of MPSH orbital energy will occur, to which electrochemical potential, *μ_L_* or *μ_R_*, and at what bias value? From the empty gap *E_P_*(*bias*) − *E_P_*(0) and zerobias <*z*_HOMO/LUMO_> and *E*_HOMO/LUMO_ (Figure 3), one can estimate the applied voltages for which the orbital energy will be equal to the electrode electrochemical potential. At those voltages (see orange/purple lines crossing light blue/red ones in Figure 3), one may expect the orbital energy to enter either the bias window or the SP region. In the former case, the orbital will start contributing to the electronic transport; thus, there will be a jump in the tunneling current at that voltage. On the contrary, in the latter case, no current jump is expected, as the orbital energy avoids the entrance into the bias window.

**Figure 4:**
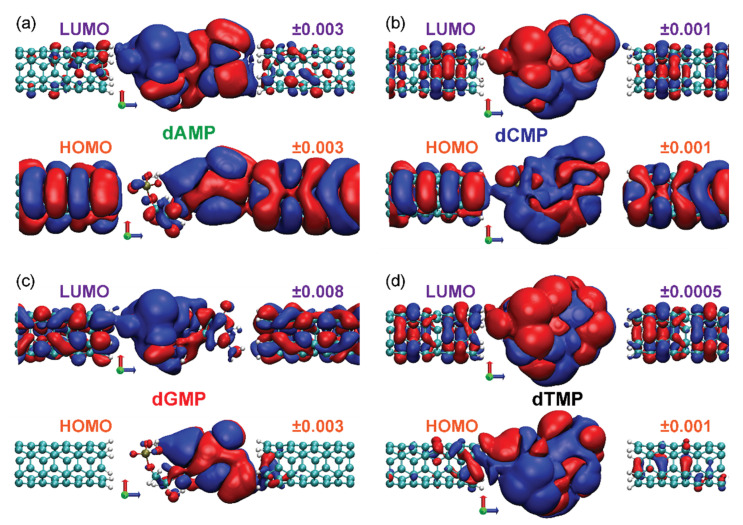
The isosurfaces of MPSH frontier orbital wave functions *ψ*_HOMO/LUMO_ calculated at zero bias for (**a**) dAMP, (**b**) dCMP, (**c**) dGMP, and (**d**) dTMP between two H-terminated CNTs. The indication of MPSH orbital (HOMO and LUMO) is above the left, with the positive (red surface) and the negative (blue surface) isovalues above the right CNTs. Red, green, and blue arrows indicate *x*, *y*, and *z* axes, respectively.

We found possible bias values at which the pinning could occur. Its occurrence depends on the MPSH orbital wave function spatial distribution. In Figure 4, we plot the HOMO and LUMO wave functions of nucleotides again in the molecular junction at lower isovalues to understand under which conditions the strong pinning will appear. The lower isovalues reveal the overlap between atomic orbitals of the molecule and CNTs, i.e., the spatial overlap of HOMO/LUMO with electrodes. The resistances on the left and the right interfaces of a molecule with electrodes depend on this overlap: a high overlap produces low resistance and vice versa. The dAMPLUMO wave function spreads across the whole molecule and, only slightly, to both CNTs (Figure 4a), as if it cannot “decide” whether to be pinned to *μ_L_* or *μ_R_*. Its energy is almost constant, never entering the SP regime but entering the bias window at both positive and negative voltages, almost symmetrically (Figure 3a). Although dGMP HOMO crosses *μ_L_* and LUMO *μ_R_* (Figure 3c), they do not pin, since they overlap with opposite CNTs (Figure 4c). The LUMO wave functions of dCMP and dTMP overlap with the left CNT (Figure 4b,d). For negative bias, their energies cross *μ_R_* to enter the bias window, while for positive voltages, they pin to *μ_L_* (Figure 3b,d). Thus, at the bias value at which the strong pinning appears, the orbital energy equals the electrochemical potential of an electrode, and its wave function spatially overlaps with the same electrode. This energy and space overlap is referred to as orbital and electrode hybridization.

### 3.4. The Tunneling Current and Rectification

**Figure 5:**
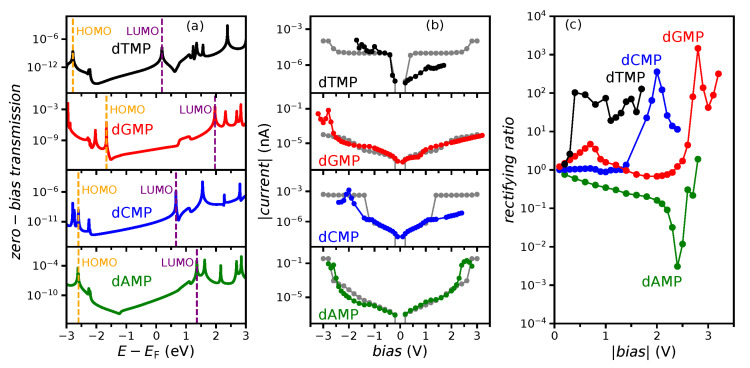
(**a**) Zero-bias transmission spectrum *T*(*E*,0), (**b**) absolute tunneling current |*I*|, and (**c**) rectifying ratio *RR* of the four nucleotides. In (**a**), orange and purple dashed lines mark the zero-bias HOMO and LUMO energies, while in (**b**), the gray curves are absolute currents obtained from the zero-bias approximation.

All of the discussed properties so far impact the tunneling current *I*, which was obtained from Equation (2) and its rectification. In Figure 5, we plotted the zero-bias transmission, the absolute tunneling current |*I*|, and the measure of rectification, the rectifying ratio, which was defined as the absolute value of the quotient of tunneling currents at negative and positive voltages, *RR =* |*I*(*bias* < 0)/*I*(*bias* > 0)|, of four nucleotides. Calculated zero-bias HOMO and LUMO energies correspond well to transmissions maxima (Figure 5a). To examine the relation of the finite-bias transport characteristics to zerobias, coupled with empty-gap features, we will now discuss the *I*-*V* and *RR* curves for all nucleotides in detail. For dAMP, the current experiences two jumps, at around −2.5 and 2.2 V (almost symmetrical green curve in Figure 5b), corresponding to entrance into the bias window of LUMO in both cases (Figure 3a). As the orbital is centered around the middle of the gap (Figure 2b) and does not spatially overlap with any of the electrodes (Figure 4a), its energy does not change significantly with bias (Figure 3a), starting to contribute to transport at almost symmetric values of positive and negative bias. However, even this slight asymmetry produces a significant dip in the *RR* curve (green line in Figure 5c) at 2.4 V. For dGMP, the abrupt change in current begins at −2.6 V (red line in Figure 5b), thanks to HOMO contribution (Figure 3c). For dGMP, LUMO also contributes to transport at voltages lower than −3.2 V (Figure 3c). Being weakly pinned, *E*_HOMO_ decreases, while *E*_LUMO_ increases at positive voltages (Figure 3c), avoiding entrance into the bias window for the studied range, contributing in that way to the significant rise in the *RR* (red curve in Figure 5c) at 2.6 V.

We will now show how the strong pinning influences the transport characteristics. For dCMP, the *I*-*V* curve (blue line in Figure 5b) is almost perfectly symmetric from −1.3 to 1.3 V. Below −1.3 V, LUMO begins contributing to the current, while above 1.3 V, it escapes the bias window after being pinned to the *μ_L_* (Figure 3b). For the bias range between the two voltages, the LUMO, centered around the middle of the gap (Figure 2b), having spatial overlap with the left electrode (Figure 4b), has almost constant energy. Therefore, the dCMP rectifying ratio (blue line in Figure 5c) is flat and equal to 1 up to 1.3 V when it suddenly increases due to the SP, i.e., the hybridization of LUMO with the left electrode. At the same voltage, the resistance at the dCMP interface with the right CNT starts to increase, while the resistance on the left remains the same. Thus, when the ratio of interfacial resistances starts to change, the rectification will occur. The discussion for dTMP is similar to dCMP. The difference is that the significant electronic transport rectification (black lines in Figure 5) appears at 0.3 instead of 1.3 V. Such a low voltage appears, since dTMP zero-bias *E*_LUMO_ is much closer to Fermi energy *E*_F_ than for any other nucleotide (Figure 3d and Figure 5a).

### 3.5. Zero-Bias Approximation

As this work deals with the possible estimation of finite-bias from zero-bias properties, we will now discuss obtaining a finite-bias tunneling current from a zero-bias transmission spectrum *T*(*E*,0) (see Computation details section), as given in Figure 5a. The gray curves in Figure 5b represent the tunneling current calculated from zero-bias approximation by inserting *T*(*E*,0) instead of *T*(*E*,*V*) in the integral in Equation (2). As expected, the absolute currents for the opposite voltages are the same, and *RR* is always equal to 1. Therefore, one cannot use it for rectification, only for the current estimation. The zero-bias approximation is typically used to save the computing time and is applicable at low voltages [41,42]. In our case, it fails even at small voltages, as for dTMP (black vs. gray curve in Figure 5b), rectification appears at 0.2 V.

### 3.6. Rules for Pinning Regimes Diagram Construction

Now, we can summarize the procedure to construct the pinning regimes diagram, i.e., the MPSH orbital energy bias dependence (Figure 3) based on empty-gap and zero-bias properties. Here, we list the steps:

1. From zero-bias transport calculation, obtain HOMO and LUMO: their energies *E*_HOMO/LUMO_, wave functions *ψ*_HOMO/LUMO_, and average *z* coordinate <*z*_HOMO/LUMO_> of atoms having the most significant contribution to the orbital (whose atomic orbital coefficients *c* from Equation (1) are higher than 0.1, for example);
2. Calculate the bias dependence of the empty-gap electrostatic potential *E_P_*(*bias*) − *E_P_*(0) at <*z*_HOMO/LUMO_> (in fact, this step does not even require finite-bias transport calculation);
3. Calculate HOMO/LUMO weak-pinning energy *E*^WP^_HOMO/LUMO_(*bias*) from Equation (3) and voltage at which it becomes equal to *μ_L_* and *μ_R_*. At these voltages, the orbital energy enters either the bias window (a possible drastic increase in the tunneling current) or the SP regime (no dramatic tunneling current changes;
4. Check the spatial overlap of the orbital with both electrodes (*ψ*_HOMO/LUMO_). The strong pinning will occur if there exists a dominant overlap (hybridization) of the orbital with the electrode whose electrochemical potential *E*^WP^_HOMO/LUMO_ intersects.

Not only do the zero-bias and empty-gap properties influence the pinning regimes diagram, but they also affect the transport characteristics. At bias values determined in step (3), significant changes in *I*-*V* and *RR* should occur.

## 4. Discussion

As a rule of thumb, if the orbital is on the left (right) compared to the gap center, its energy would increase (decrease) with the increment of bias, with the variation more pronounced if the orbital location is further from the gap center (Figure 2b and Figure 3). One could observe such behavior in other works [19,20,29]. The energy variation of orbitals of DNA nucleotides within the graphene−hBN heterostructure nanogap [19] complies with this rule of thumb. For example, looking at Figure 7 of Reference [19], one can notice that selected orbitals (A1, T2, G1, and C1), located on the molecule, are positioned left compared to the gap center. The energy of the orbitals more to the left (A1 and G1) has a noticeable linear increase with bias, while for the other two (T2 and C1), closer to the gap center, this variation is not pronounced. The bias variation of energies of orbitals (G1, G2, A2, and C2) located on DNA nucleotides in a double-functionalized graphene nanogap, given in Figure 3 of Reference 20, also follows the rule of thumb.

Although our work concerns a model system (a molecule in a nanogap between two side-embedded nanoelectrodes around a nanopore), the presented results may influence expanding fields of DNA and protein nanotechnologies [3,4,43]. Recent progress in DNA manipulation of translocation through solid-state nanopores [44] and on surfaces [45] by atomic force microscopy (AFM) could lead to the realization of sequencing, both of DNA and proteins. Extreme mechanical deformations, i.e., compression of folded proteins, could significantly change their transport properties [46]. One would expect a stretching of DNA or protein chain in the case of AFM control of translocation through a nanopore. In our previous work, we found a low influence of neighboring nucleotides (from single-strand DNA) on the transport properties of the nucleotide in the nanogap [18]. Thus, we expect no significant effects on the transversal tunneling transport of stretching deformations caused by AFM-controlled DNA and protein translocation through a nanopore. Concerning DNA sequencing based on tunneling transport through nucleotides between transversal electrodes, previous works [11,18] show that thermal fluctuations (studied as a variation of molecular position within a nanogap) may influence the electronic properties of a tunneling current and current rectification. However, the distinction between nucleobases would still be possible using these parameters. The rectification turns out to be a more reliable parameter less sensitive to fluctuations than the current [18].

We believe it is significant to mention a recent experimental study of 100-base-pair double-stranded DNA connected to gold leads at a 30 nm distance [47], regardless of the conceptual difference compared with our setup. It shows that longitudinal electronic transport proceeds through the backbone via the hopping mechanism. In our case, the transport current is not sequential but coherent (tunneling), since the distance between CNT electrodes is around 1.5 nm and happens through a molecular level localized on a single nucleotide. Moreover, in our case, no covalent bonds exist between a molecule and the electrodes.

That is the main difference between the single-molecule rectifiers and the system studied here, as mentioned in the Introduction. Still, rectification appears in both cases. In the case of standard rectifiers, a set of rules for obtaining desirable rectification through the control of frontier orbital properties is established [30,31,48]. As we will show, some of the rules are satisfied in our study, indicating that they are valuable guidelines for device design even in the absence of covalent bonding. The first two rules recommend using asymmetrical anchoring groups [30,48,49] for promoting HOMO and LUMO energy alignment with *E*_F_, which would produce the strong pinning of both levels to electrochemical potentials of corresponding electrodes. For DNA and protein sequencing devices, the alignment of frontier orbital energies and pinning could be achieved using asymmetric electrodes [20] or proper electrode functionalization [40], as the use of anchoring groups to align HOMO and LUMO to Fermi energy seems too challenging. We chose a simple system with symmetric electrodes to emphasize the studied properties. The hydrogen termination of CNTs promotes LUMO alignment [40]. Thus, for all nucleotides, the orbital with a principal contribution to transport is LUMO. It has the most impact on dTMP current and rectification (Figure 5b,c) since its energy is closest to *E*_F_ at zero bias (Figure 3d and Figure 5a). The second two rules advise the spatial decoupling of HOMO and LUMO [30,48,50] and bias-dependent charge transfer from one to another [31]. In DNA and protein sequencing applications, one cannot influence the spatial decoupling of frontier orbitals. The nucleotide with the highest spatial decoupling of HOMO and LUMO is dGMP, which is reflected as significant weak-pinning bias dependencies of their energies, which leads to energy gap change (Figure 3c). We found no nucleotide with bias-dependent charge transfer from HOMO to LUMO (the fourth rule [31]) in the studied setup.

## 5. Conclusions

In this work, we studied the electronic transport properties of DNA nucleotides between two H-terminated carbon nanotube electrodes using DFT and NEGF at finite bias. The bias dependence of frontier orbital energies that manifests in two distinctregimes, the weak and the strong pinning, are described. Furthermore, the tunneling current and its rectification expressed as the rectifying ratio *RR* were calculated. We established a procedure to estimate the finite-bias dependence of HOMO and LUMO energies, i.e., the pinning regimes diagram (Figure 3), from zero-bias and empty-gap properties: spatial distribution of HOMO and LUMO wave functions, their energy and spatial overlap with electrodes, the electrostatic potential of the empty gap, and molecular position inside the gap. The crucial points in the diagram are the intersections of estimated weak-pinning energies with electrochemical potentials of electrodes. At these points (voltages), orbital energy either enters or starts avoiding the bias window, corresponding to staying in the weak or transiting to the strong pinning regime. The strong pinning will occur if there is a dominant hybridization (in energy and real space) of the orbital with the electrode whose electrochemical potential its energy intersects. Upon entering the bias window, the tunneling current may increase drastically, while if it starts avoiding it, no such change is expected. Thus, at the same points (voltages), the tunneling transport (*I*-*V* and *RR*) is most likely to undergo significant changes. In our setup, there are no covalent bonds between nucleotides and CNTs (electrodes). Still, the rules defined for single-molecule rectifiers could serve as valuable guidelines for designing devices similar to one studied here.
